# Skin Cell Heterogeneity in Development, Wound Healing, and Cancer

**DOI:** 10.1016/j.tcb.2018.05.002

**Published:** 2018-09

**Authors:** Emanuel Rognoni, Fiona M. Watt

**Affiliations:** 1 King’s College London, Centre for Stem Cells and Regenerative Medicine, 28th Floor, Tower Wing, Guy’s Hospital Campus, Great Maze Pond, London SE1 9RT, UK

**Keywords:** stem cell plasticity, fibroblast lineages, microniches, wound healing, skin cancer

## Abstract

Skin architecture and function depend on diverse populations of epidermal cells and dermal fibroblasts. Reciprocal communication between the epidermis and dermis plays a key role in skin development, homeostasis and repair. While several stem cell populations have been identified in the epidermis with distinct locations and functions, it is now recognised that there is additional heterogeneity within the mesenchymal cells of the dermis. Here, we discuss recent insights into how these distinct cell populations are maintained and coordinated during development, homeostasis, and wound healing. We highlight the importance of the local environment, or niche, in cellular plasticity. We also discuss new mechanisms that have been identified as influencing wound repair and cancer progression.

## Highlights

Different epidermal and dermal cell populations not only contribute to tissue homeostasis but also to wound repair and underlie tumour heterogeneity and progression.

Skin cellular behaviour and fate are regulated by interactions at the level of single cells, termed microniches, as well as on higher scales.

Individual niche components and transcription factors can be identified that significantly affect stem cell maintenance and behaviour.

Cell population-specific mechanisms regulate cellular plasticity and behaviour during wound repair and tumour development.

Epidermal stem cell plasticity is achieved by a loss of lineage specificity during wound healing, and may become permanent in skin cancer.

## Skin Architecture

**Figure 1:**
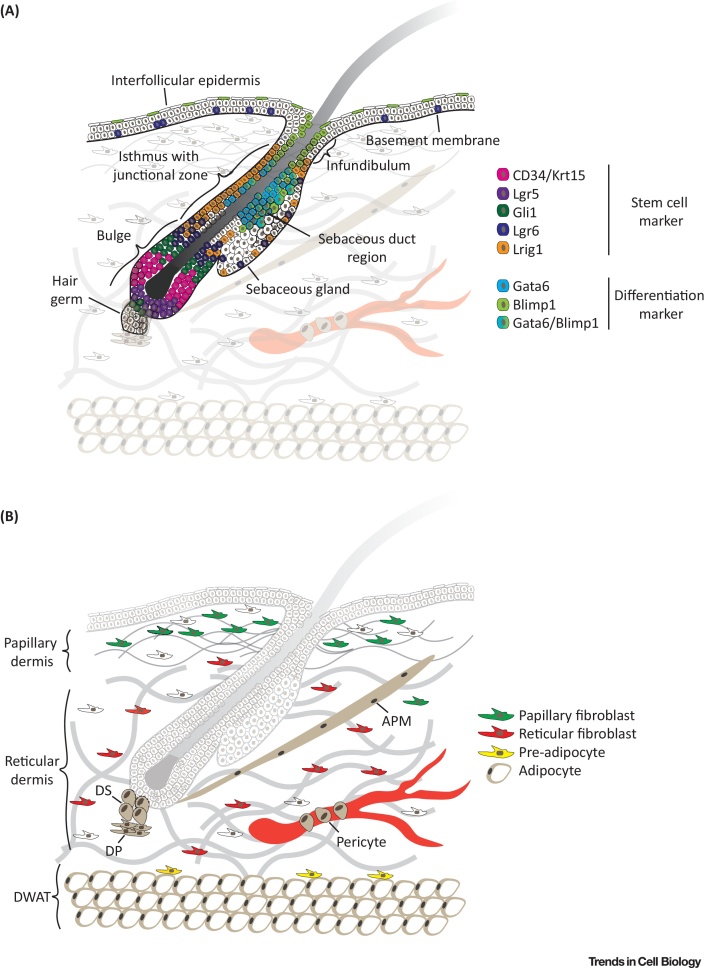
Stem Cell and Mesenchymal Cell Population Diversity in the Skin. (A) Stem cell and other cell populations in the interfollicular epidermis and hair follicle. Distinct markers shown in the colour code legend have been identified mapping the spatial distribution of distinct stem cell and differentiated cell populations in the hair follicle bulge, isthmus, infundibulum, sebaceous gland, and interfollicular epidermis. A basement membrane separates the epidermis from the dermis. Note that the sebacous duct is not visible. (B) Mesenchymal cell populations in the indicated dermal layer. **Papillary fibroblasts** are located close to the basement membrane and surrounded by thin collagen fibres whereas **reticular fibroblasts** reside in the central dermis associated with thick collagen bundles (grey). The preadipocytes are located close to the DWAT, which harbours the mature, lipid-filled adipocytes. In addition, the dermis contains specialized fibroblast subpopulations (brown) forming the DP, DS, and APM, as well as surrounding the blood vessels (pericytes). Abbreviations: APM, arrector pili muscle; DP, dermal papilla; DS, dermal sheath; DWAT, dermal white adipose tissue; ECM, extracellular matrix; Lrig1, leucine-rich repeats and immunoglobulin-like domains protein 1.

The skin consists of two layers, the upper epidermis and the lower dermis, which are separated by a basement membrane, and harbours specialised structures such as hair follicles (HFs) and sweat glands (Figure 1). The epidermis is a multilayered stratified epithelium, which is constantly renewed throughout life; homeostasis is maintained by a balance between basal cell proliferation and suprabasal cell differentiation/stratification. At the onset of differentiation, basal cells become detached from the basement membrane, stop proliferating, and once located in the suprabasal cell layer, start executing terminal cell differentiation programmes culminating in loss of the nucleus. The precise sequence of events for cell commitment and differentiation on the single cell and tissue scale is still being investigated (reviewed in [1]).

The dermis is composed of different sublayers that are distinguished by cell type, cell density, and extracellular matrix (ECM) composition (reviewed in 2, 3). The papillary layer is located close to the basement membrane and shows a high fibroblast density and dense meshwork of thin, poorly oriented collagen fibres. The reticular dermis is the central and largest layer of the dermis, consisting of thick, highly organised collagen fibre bundles and lower cellular density. Under the reticular dermis lies the dermal white adipose tissue (DWAT), also referred to as hypodermis, which harbours pre- and mature adipocytes. Mature adipocytes are filled with lipids (Figure 1B). In addition there are specialized fibroblast subsets in the skin that form the **dermal papilla** (DP) (see Glossary) at the base of HFs, the **dermal sheath** that is wrapped around HFs and the **arrector pili muscle** (APM), which is connected close to the HF **bulge** and is responsible for piloerection (reviewed in [4]).

In this review we highlight recent advances in dissecting different cell subpopulations in the epidermis and dermis. We discuss how epidermal and dermal cells interact with each other during homeostasis, wound healing, and cancer.

## Stem Cell Populations in the Epidermis

Multiple epithelial stem cell (SC) populations have been shown to contribute to skin homeostasis. In mice a highly diverse pool of SCs has been identified in the HF, ranging from the lower HF (Lgr5^+^ and CD34^+^/Krt15^+^) bulge to the upper HF (Gli1^+^ and Lgr6^+^) and **junctional zone** (Lrig1^+^) (Figure 1A). In undamaged skin the different cells populate distinct and restricted areas in the HF, whereas upon tissue injury these cells have the intrinsic ability to give rise to all epidermal cells, including the **interfollicular epidermis** (IFE), which is located between HFs and comprises the largest pool of keratinocytes in the skin 5, 6. How these differential cellular behaviours are regulated at the molecular level is currently being investigated (Box 1).

**Figure I:**
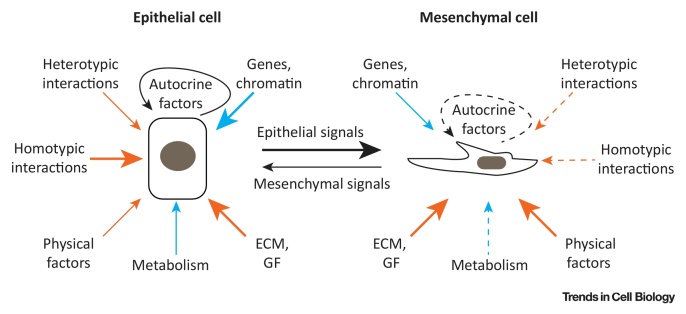
Comparison of Extrinsic and Intrinsic Regulatory Factors of Epithelial and Mesenchymal Cells in the Skin. Note that the arrow type and thickness indicates differential impact of extrinsic (orange) and intrinsic (blue) factors on epithelial (left) and mesenchymal (right) cells. Niche factors have been shown to exert a strong impact on all cell populations (solid thick arrow), impact on some cell populations (solid thin arrow), or weak/unclear/unknown impact (dashed thin arrow). A combination of extrinsic and intrinsic niche factors (black) defines the autocrine and epithelial/mesenchymal signals. Cell–cell interactions are categorised as homotypic (between neighbouring cells of the same type) and heterotypic (between other cell types such as immune, endothelial, or neuronal cells). Physical factors include tension, compression, shear stress, as well as temperature. Gene and chromatin refer to intrinsic changes in gene expression and chromatin state. Abbreviations: ECM, extracellular matrix; GF, growth factor.

Box 1 **Epithelial SC Niche Interactions**

In the IFE and HF maintenance of distinct SC populations relies on a tight interplay of intrinsic factors and extrinsic factors that define the cellular microenvironment or niche (Figure I). The composition of the niche – neighbouring cells, ECM, GF, and physical parameters – is highly location and cell specific. Key signalling pathways involved in cell microenvironment regulation in the skin include WNT/β-catenin, TGFβ, BMP, fibroblast growth factor, SHH, and Notch, which can act in an autocrine and paracrine manner 74, 75, 76.

Key intrinsic factors include SC-specific regulation of metabolism, gene expression, and chromatin arrangement. For example, HFSCs produce more lactate than other epidermal cells, suggesting that differences in cellular glycolytic metabolism influence SC activity [77]. In the HF bulge the transcription factor Foxc1 is specifically induced in HFSC upon activation of the hair cycle, and regulates the expression of BMP and nuclear factor of activated T cells 1 to maintain HFSC quiescence during HF growth 78, 79.

Within the niche interactions between neighbouring cells are important regulators of cell behaviour. Upon epithelial SC ablation neighbouring cells are able to replace niche SC even if they are committed progenitors [73]. In addition, loss of single ECM components can be sufficient to dramatically impact SC maintenance in the niche. DNA damage induced proteolysis of collagen 17a1 in the HF bulge with age leads to a cyclic elimination of HFSCs through epidermal terminal differentiation [80]. Maintaining or replenishing collagen 17a1 in the skin inhibits the ageing phenotypes, suggesting a possible therapeutic application 80, 81.

Extrinsic niche factors can act on a single cell level and are either produced by the SCs themselves [82] or provided by surrounding cells such as progenitor cells, fibroblasts, resident immune cells, or sensory neurons 39, 40, 83, 84, 85. Sensory neurons in contact with the HF create a perineural niche microenvironment in the bulge and isthmus. The secretion of SHH results in a specialised subset of bulge cells characterised by high Gli1 expression and activated Hedgehog signalling [83]. A subset of resident regulatory T cells located close to the HF bulge and expressing high levels of Jag1 promotes HFSC proliferation and differentiation through direct activation of Notch signalling in HFSCs [84]. Impaired crosstalk between epidermal and dendritic T cells severely affects wound repair in aged skin, highlighting the importance of different resident immune cell types in homeostasis as well as tissue repair [85].

Physical parameters such as tension, pressure, or temperature contribute to the cellular microenvironment, are sensed by distinct cell populations, and influence cellular fate. For example, in epidermal SCs a mechanosensory complex consisting of emergin, nonmyosin-IIA, and actin affects gene transcription and lineage commitment [86]. Extrinsic force-induced relocation of emergin and a change in cellular G-actin levels leads to a switch in histone methylation and loss of heterochromatin anchoring to the nuclear lamina, thereby inhibiting global gene transcription. It has also been noted in single cells that there is a potential interplay between the actin cytoskeleton and epigenetic modifications [87].

It remains to be determined whether force-induced changes in chromatin structure are transient or if SCs functionally adapt and memorise the stress response. It was recently shown that epithelial SCs exhibit a memory of previous inflammation upon tissue damage by maintaining chromosomal accessibility at key stress response genes, promoting faster transcription [88]. This memory does not require the presence of resident immune cells but is critically dependent on Aim2 (absent in melanoma 2), which is an activator of the inflammasome upstream of caspase-1 and interleukin-1β. A side effect of this increased responsiveness to stressors in epithelial SCs might be increased susceptibility to autoimmune and hyperproliferative disorders including cancer.

Epidermal β-catenin activation not only results in cell-intrinsic effects but also alters the behaviour of neighbouring cells [89]. Recent single cell gene expression profiling has shown on cell intrinsic β-catenin activation, gene expression heterogeneity is reduced in neighbouring cells and the effect is most dramatic for genes associated with protein synthesis [90]. The effect is dependent on cell–cell contact and the changes in gene expression are accompanied by a shift to a more proliferative SC state.

It is conceivable that many more specialized SC subpopulations, maintained and defined by a complex array of niche factors, will be discovered; indeed that all epidermal cells in contact with ECM have SC properties.

Alt-text: Box 1

During development, the epidermis is formed by a flat single layered epithelium known as the surface ectoderm. Local induction of Wnt signalling in the epidermis and subsequently in the dermis leads to the formation of **HF placodes**, which are characterised by expression of adult SC markers such as Sox9 [7]. While adult skin SC maintenance and differentiation depend on signals from local **niches** such as the HF bulge, during hair bud development, SC specification is achieved by asymmetric cell division, differential levels of Wnt signalling, and the response to sonic hedgehog (SHH) of basal and suprabasal cells [8]. During further maturation of the HF placode, the SC markers start to segregate into the distinct HF structures including the HF bulge **isthmus** and **sebaceous gland** (reviewed in [9]). When the HFs form they further provide niche structure and signals to induce the specification of Merkel cells, a specialised subpopulation of keratinocytes that become organised into touch domes around primary HFs. These innervated mechanoreceptors mediate light touch sensation [10].

Beside epithelial cells (keratinocytes and Merkel cells), the adult IFE harbours melanocytes and immune cells (Langerhans cells and γδT-cells). Until recently the organisation of epithelial SCs within adult mouse IFE was a matter of some debate. Initial *in vivo* clonal analysis in mouse tail and ear epidermis indicated that a single cell population is responsible for epidermal homeostasis, and variation in clone size could be explained by stochastic (random) cell division of a homogeneous population of keratinocytes, referred as the **neutral drift model** 11, 12, 13. However, it is now clear that IFE SCs are heterogeneous. More detailed characterisation of tail IFE, in which clonal growth studies supporting the neutral drift model were carried out, revealed that there are two distinct pathways of terminal differentiation, one corresponding to the **parakeratotic** scale IFE that is not associated with HFs, and the other to the **orthokeratotic** interscale IFE located close to the HFs, each being generated and maintained by a different pool of basal cells 14, 15. The size of the scale and interscale regions is controlled by epidermal Eda and Wnt/β-catenin signalling, and there is a corresponding patterning of melanocytes and papillary dermal fibroblasts [14]. Lgr6^+^ cells contribute to the interscale but not to the scale IFE [5]. The scale and interscale IFE can also be distinguished by expression of Slc1a3 and Dlx1, respectively [16]. The two tail IFE SC compartments differ in their proliferative dynamics, gene-expression profiles and ability to repair the epidermis after injury 16, 17.

Single cell transcriptomic analysis of mouse dorsal epidermis and cultured human epidermis has identified at least two distinct IFE SC transcriptional signatures, even though there appears to be a single terminal differentiation programme 18, 19. It is not known at present whether the cellular heterogeneity in the IFE reflects differential susceptibility to initiating keratinocyte differentiation. In addition the proliferative properties of cells in the IFE basal layer are influenced by the HF cycle. **Lineage tracing** experiments have revealed that while cell clones associated with HF show a rapid increase in size during the HF growth phase, distant clones cycle more slowly, yet can be mobilised upon tissue injury [20]. Thus, while in mouse tail IFE, distinct SC populations are associated with unique differentiation programmes, SC heterogeneity in mouse back skin IFE underlies a single differentiation programme and could reflect different **cellular states**.

To gain further insights into the proliferative dynamics of epidermal cells with age, in recent yearsclonal analysis has been applied to human epidermis by making use of sunlight induced mutations in cancer-associated genes, such as p53, as markers 21, 22. This has led to conflicting conclusions about the relative importance of positive selection and neutral drift in clonal evolution. Recently, by sequencing larger areas of skin than previously and focusing on skin from patients who had previously developed a skin tumour, it has been possible to establish that some human mutant clones are too large to be accounted for solely by neutral drift. Rather, secondary mutations arising at the edge of a mutant clone have a selective growth advantage [23].

## Mesenchymal Cell Heterogeneity and Behaviour in Dermal Homeostasis

Beside its role as an ECM-rich scaffold, the dermis harbours highly diverse fibroblast, **pericyte**, and immune and endothelial cell populations that dynamically change with age and influence the properties and cellular behaviour of the overlying epidermis 2, 4, 24 (Figure 1B). Although the dermal layers can be easily distinguished by collagen structure and cellular density, the cellular events generating and maintaining dermal architecture have not been explored in detail until recently. During mouse embryonic development, dermal fibroblasts arise from at least two spatially and functionally distinct **cell lineages** that differentiate into distinct subpopulations and contribute to the dermal layers 25, 26. Neonatal dermis fibroblasts of the papillary layer are characterised by active Wnt signalling and proliferation, whereas populations in the reticular layer show increased expression of ECM and immune cell associated genes 26, 27, 28. Whether bone-marrow-derived mesenchymal stromal cells (MSCs) contribute to the resident fibroblasts of mouse dorsal skin under homeostatic conditions or following wounding is controversial (reviewed in [2]).

During development, gene expression in dermal fibroblasts is highly dynamic and there is a swift change in dermal fibroblast behaviour on the tissue scale during dermal maturation 26, 27. While fibroblasts are highly proliferative during embryonic development they rapidly stop proliferating in the postnatal dermal growth phase, which is characterised by extensive ECM deposition and remodelling. Clonal analysis reveals that individual fibroblasts within clones start to be segregated by increased ECM deposition, leading to a dramatic reduction in fibroblast density postnatally [27]. In addition there is an increase in the adipocyte layer with age [29]. The different dermal fibroblast lineages are spatially segregated in P2 mouse skin, whereas mixing of lineages occurs during subsequent dermal maturation. Recent single cell RNA sequencing studies of human dermal fibroblasts identified several transcriptionally distinct subpopulations, some of which are spatially segregated whereas others are not 28, 30. Further studies are needed to clarify whether the transcriptionally heterogeneous fibroblast subpopulations in human dermis represent different cellular states or functionally distinct fibroblast lineages.

While the nature of the switch between fibroblast proliferation and ECM production is unknown, gene expression analysis of neonatal (proliferative) and aged (nondividing) dermal fibroblasts suggests that it is controlled by epigenetic changes at the chromatin level 27, 31. *In vitro* studies suggest that there is not a common nonproliferative state in fibroblasts but that it is rather an accumulation of different states that are actively maintained 32, 33. Besides actively reinforcing the nondividing cell cycle state and repressing the transition into senescence or terminal differentiation, aged fibroblasts remain highly metabolically active [34]. They increase expression of ECM proteins such as collagen I and III, which is partly due to changes in expression of miRNAs such as miR-29 [35].

## Epidermal–Dermal Interactions via Reciprocal Niche Signals

Reciprocal signalling between epidermis and dermis plays a key role in skin development, homeostasis, wound repair, and cancer. A prime example of tight temporal and spatial regulation involves the DP fibroblasts at the base of the HF and the overlying HF bulb (reviewed in 36, 37). The transcriptional repressor Blimp1 is a key target and mediator of this interaction [38]. Upon HF induction during development epidermal Wnt/β-catenin signalling induces Blimp1 expression in the DP via transforming growth factor (TGF)β signalling. In the DP, Blimp1 promotes Wnt/β-catenin signalling activity and HF growth.

When the DP matures during HF growth, heterogeneity of mesenchymal signals at the single cell level involving gradients of Wnt ligands and bone morphogenetic protein (BMP) inhibitors creates distinct microniches along the epithelial–mesenchymal interface in the DP [39]. By secreting distinct combinations of factors these microniches coordinate the hierarchy of self-renewal and differentiation states of epithelial cells in the HF matrix, enabling formation of the different HF layers.

The HF bulge SC niche harbours a distinct combination of ECM components. Bulge cells have been shown to create microniches for APM cells by depositing specific ECM components including nephronectin in the basement membrane [40]. Nephronectin is specifically recognised by α8β1-integrin positive mesenchymal cells of the APM, which is essential for proper anchorage and function. Interestingly, regenerated HFs in wounds lack APMs, indicating that additional factors or specific mesenchymal subpopulations are required for APM formation 26, 27.

In contrast to the HF, in the IFE the spatial and temporal coordination of epidermal–dermal interactions has not been defined at single cell resolution. However, in tail skin, leucine-rich repeats and immunoglobulin-like domains protein 1 (Lrig1) expression is selectively upregulated in dermal fibroblasts that underlie the interscale epidermal compartment, pointing to different dermal niches in the interscale and scale [14].

One of the key pathways of epidermal–dermal communication is via canonical Wnt signalling. Activation of Wnt/β-catenin in basal keratinocytes of adult mouse epidermis induces a rapid increase in fibroblast proliferation and stimulates ECM remodelling 31, 41.

It seems likely that, similar to the epidermis, dermal subpopulations are maintained by a complex combination of extrinsic and intrinsic factors (Box 1). On the one hand, reciprocal transplant experiments involving fibroblasts of different developmental origins (oral cavity vs skin) have revealed that their differential ECM deposition and migratory behaviour is maintained and thus are cell intrinsic features [25]. Furthermore Sox2^+^ DP fibroblasts maintain their identity in cell culture [42]. On the other hand, different fibroblast subpopulations respond to distinct paracrine signals from the epidermis. Epidermal SHH signalling stimulates proliferation and ECM remodelling of the upper dermis, while TGFβ-2 influences proliferation, ECM deposition and differentiation of the lower dermis [41]. In addition, secreted factors of Wnt/β-catenin activated keratinocytes promote adipocyte differentiation and expansion of the hypodermis [29] whereas PDGF expressed by preadipocytes stimulates HF SC activity [43].

## Epidermal SC Behaviour during Wound Healing

**Figure 2:**
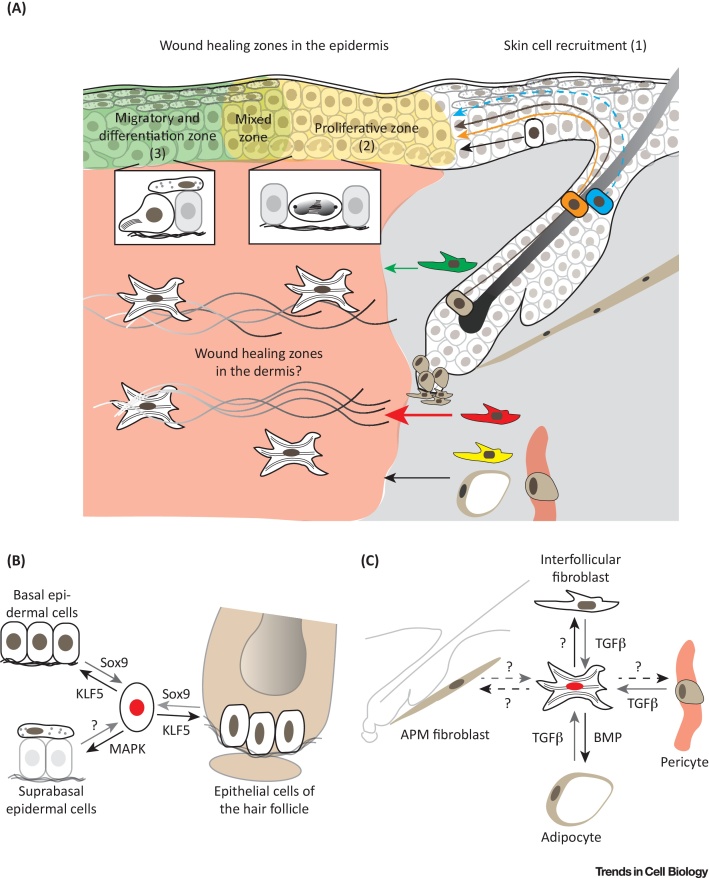
Epidermal and Mesenchymal Cell Heterogeneity and Plasticity During Wound Healing. (A) Stem cells and other cell subpopulations are recruited during wound healing in the epidermis and dermis. While cell lineages of the bulge (brown), infundibulum (orange), and interfollicular epidermis (white) enter more as a cohesive cell population (solid arrows), sebaceous duct cells (blue) migrate to the wound site suprabasally as individual cells (dashed arrow) (1). Note that once different cells of the hair follicle and interfollicular epidermis enter the wound healing zones they exhibit similar cellular behaviours in proliferation (2) and cell migration and differentiation (3). Wound healing zone key features are shown in boxes,with cell division in the proliferative zone (2) and cell migration and differentiation in the migratory and differentiation zone (3). In the mixed zone all key features can be observed. Whether these zones of collective behaviour propagate into the dermis and how mesenchymal cells are organised during wound healing are less clear. During wound healing reticular fibroblasts (red) are the first and most abundant fibroblasts to enter the wound bed (red thick arrow) and are the major source of myofibroblasts (white cell in the wound bed). Papillary fibroblasts (green) enter the wound bed at a later stage (green thin arrow). Preadipocytes (yellow), adipocytes and pericytes (brown) have also been shown to contribute to dermal wound healing (black thin arrow). (B) Plasticity of epithelial cells during wound healing, with associated key signalling pathways and transcription factors. Epithelial cell populations of the interfollicular epidermis and hair follicle transiently lose their lineage identity during wound healing (central cell with red nucleus) and are able to differentiate (black arrow) or dedifferentiate (grey arrow) and acquire the potential to regenerate all tissue structures. (C) Plasticity of mesenchymal cells during wound healing with associated key signalling pathways. Mesenchymal cells close to the wound bed become activated (grey arrow), are referred to as myofibroblasts, and change their behaviour and transcriptional programme (central cell with red nucleus). Whether APM fibroblasts also participate is unknown (dashed grey arrow). During the wound resolution phase myofibroblasts are able to convert to adipocytes, **interfollicular fibroblasts** (solid black arrow) and presumably also other cell populations (dashed black arrow). Abbreviations: APM, arrector pili muscle; BMP, bone morphogenetic protein; MAPK, mitogen-activated protein kinase; TGFβ, transforming growth factor β.

Wound healing and tissue regeneration are coordinated processes that involve epidermal, dermal, endothelial and immune cells, and can be divided into distinct phases (reviewed in 44, 45). Upon wounding, a blood clot forms and immune cells infiltrate the wound site (inflammatory phase). The cells from the epidermis and dermis start proliferating and migrate into the wound bed to close the wound (proliferative phase). Then, dermal cells deposit and restructure the ECM in the wound bed (resolution phase). Of note, wound healing responses differ between species: for example, in mice wounds close mainly through tissue contraction as a result of the action of the **panniculus carnosus muscle**, a structure that is absent in human skin (reviewed in [46]). In mouse skin, several epidermal cell populations contribute to the skin wound repair process, leading to the picture that not only cells close to the wound edge but also HF epithelial cells are recruited to the wound site (Figure 2A,B). How distinct cell populations from different niches coordinate on the tissue scale and move into the wound bed, ensuring rapid wound closure and tissue regeneration, has only recently been addressed.

In the past, two distinct mechanisms for epidermal cell movement into the wound bed have been proposed. One involves a smooth flow of epidermis following a homeostatic rule of unidirectional basal to suprabasal transfer of cells with front edge movement that is achieved by basal cells migrating across the wound 47, 48. The second, referred to as leapfrogging, involves suprabasal cells sliding over the leading basal cells to become basal cells themselves 49, 50. Two recent studies, combining *in vivo* live cell imaging, lineage tracing, and transcriptomic analysis, have mapped the anatomy and spatiotemporal dynamics of the wound healing response and identified two concentric zones of cellular activity and differential gene expression 51, 52. The cell population closest to the wound edge is characterised by rapid migration and differentiation. Further from the wound there is a zone with high epidermal proliferation and little migration along the basement membrane. In the first (migratory) zone both basal and suprabasal cells actively migrate with increasing speed towards the wound centre and upregulate genes involved in ECM remodelling and cell adhesion. These include integrin α5β1, a fibronectin receptor, which enables keratinocytes to migrate on the provisional ECM deposited by immune and fibroblast cells. Epidermal cell migration and differentiation rates are coupled, inducing a coordinated tissue thickening over time in the leading edge. The second (proliferative) zone of keratinocytes not only supplies new cells but also controls the involvement of the surrounding unwounded epithelium during re-epithelialisation. During wound healing, the direction of cell movement within the migratory epidermal zone influences cell division orientation towards the wound bed centre, demonstrating a spatial interplay between proliferation and migration. Clonal lineage tracing reveals that most of the committed progenitor cells become highly proliferative and rapidly differentiate in the early wound healing phase, whereas basal (SC-enriched) cells become activated and increase the pool of SCs later. These studies suggest a model whereby the nonproliferating leading edge functions as a scaffold, preparing the wound bed for efficient repopulation towards the wound bed centre and protecting SCs during tissue repair. The clonal dynamics of different SC populations from the HF and IFE are similar, indicating that the behaviour balancing proliferation and differentiation within the wound healing zones is independent of the original cell. Furthermore, the leading edge cell gene signature is specified independently of cell division or inflammation. This indicates that both intrinsic and extrinsic factors establish the wound healing zones.

Epidermal wound healing induces plasticity in differentiated sebaceous duct cells such that they **dedifferentiate**, proliferate, and contribute to long-term maintenance of the IFE [53]. These cells are located in the suprabasal layer of the sebaceous duct that connects the HF junctional zone with the sebaceous gland and are defined by high expression of the transcription factor GATA6, a key regulator of the sebaceous duct lineage during homeostasis [53]. Upon wounding, differentiated GATA6 lineage cells in the HF duct become mobilised, migrate to the wound site suprabasally as individual cells, reattach to the basement membrane, and start proliferating to regenerate the IFE in the wound bed [53]. Mitogen-activated protein kinase signalling is a key pathway that regulates keratinocyte differentiation, while the signals regulating dedifferentiation remain unclear 53, 72. *In vivo* live imaging reveals that downward cell migration is more pronounced at the wound edge than distally. This is in contrast to the behaviour of basal Lrig1^+^ cells of the HF junctional zone, which enter the wound bed as a cohesive basal cell population 6, 51, 53. GATA6 lineage cells not only contribute to wound repair but also populate the lower HF and sebaceous gland of HF close to the wound [53]. Similar cell displacement has been observed in regenerating tail IFE. Upon wounding, SC populations in the interscale and scale migrate into neighbouring territories and contribute to tissue repair outside their normal niche [16]. This observation indicates a loss of lineage restriction during tissue repair, which is controlled by the transcription factors KLF5 and Sox9 [54].

## Mesenchymal Cell Dynamics during Wound Repair and Fibrosis

Our understanding of epidermal wound healing has rapidly advanced, while the contribution of different dermal cell populations has been less clear (Figure 2A,C). Fibroblasts in the vicinity of a wound are known to become rapidly activated, are referred to as myofibroblasts, and change their behaviour and transcriptional program. TGFβ signalling is the key pathway for fibroblast activation 26, 55, 58, 61. During the whole wound repair process there is an intensive and highly dynamic crosstalk with multiple immune cell populations, which influences mesenchymal cell behaviour (reviewed in 44, 45). We and others have shown that distinct fibroblast populations show differential recruitment and contribution to wound healing 25, 26, 27. Fibroblasts residing in the reticular dermis mediate the first phase of wound repair and are the major source of myofibroblasts, whereas fibroblasts in the papillary dermis enter the wound at a later stage and are essential for HF regeneration. Moreover, while fibroblasts from the DP do not contribute to wound repair, preadipocytes and adipocytes do contribute 56, 57. Intriguingly, depletion or manipulation of specific mesenchymal subpopulations in the dermis has been shown to significantly reduce tissue fibrosis or scar formation upon tissue injury 25, 55.

Fibroblast-specific inhibition of Wnt/β-catenin signalling in mice enhances HF regeneration by preventing the early expansion of lower dermal cells [27]. In contrast, sustained Wnt/β-catenin signalling in dermal fibroblasts impairs regeneration, induces fibrotic lesions in adult skin, and inhibits adipocyte differentiation 59, 60. Lineage tracing experiments reveal that this is due to a TGFβ-mediated conversion of adipocytes to myofibroblasts [58]. Conversely, during wound healing, BMP signalling from regenerating HFs induces myofibroblasts to convert to adipocytes in the wound bed [61]. The signals promoting myofibroblast conversion to other mesenchymal cell populations are currently unclear.

## Epithelial Plasticity in Cancer

**Figure 3:**
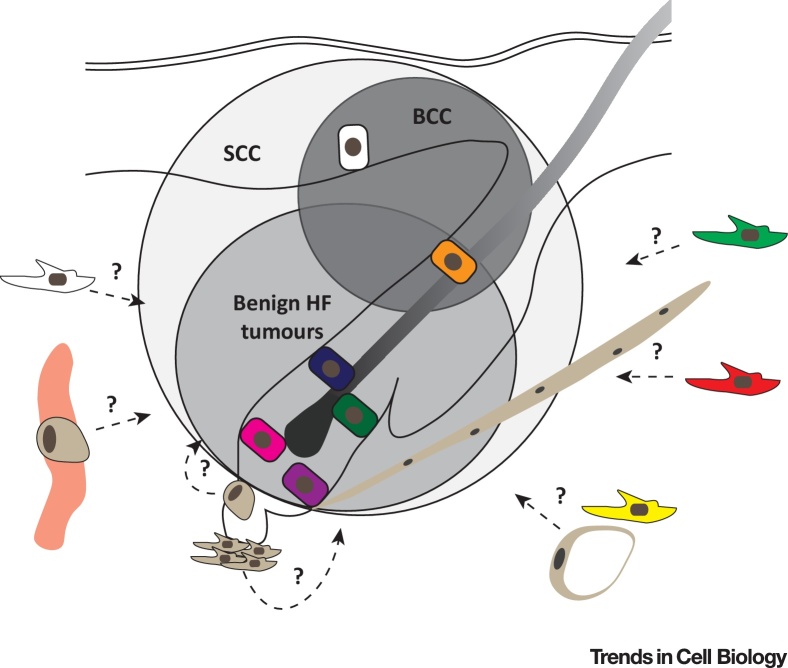
Epidermal and Mesenchymal Cell Heterogeneity in Cancer. Distinct epithelial cell populations give rise to different tumour types. Oncogenic β-catenin signalling in different hair follicle stem cell populations of the bulge (CD34/Krt15^+^, pink; Gli1^+^, green; Lgr5^+^, violet, and Lgr6^+^, dark blue) and isthmus junctional zone (Lirg1^+^, orange) induce different types of benign hair follicle tumours (grey circle), while BCCs only arise from basal (Krt14^+^) interfollicular epidermis (white cell) and Lrig1^+^ cells of the hair follicle isthmus and infundibulum (orange cell) upon Hedgehog signalling activation (dark grey circle). In contrast, a hyperactive Kras mutation is able to induce SCC in all epidermal lineages (light grey circle). If different tumour types are associated with distinct mesenchymal subpopulations (green, papillary fibroblast; red, reticular fibroblast; yellow, preadipocyte; white, undefined fibroblast; brown, APM, dermal sheath, dermal papilla fibroblast, pericyte and adipocyte) (dashed arrows) and if mesenchymal subpopulation specific signalling pathways are involved (?), is unclear. Abbreviations: APM, arrector pili muscle; BCC, basal cell carcinoma; HF, hair follicle; SCC, squamous cell carcinoma.

Similar to wound healing, different epidermal and dermal populations contribute to cancer in different ways (Figure 3). This is well illustrated in the case of oncogenic β-catenin signalling, where depending on the epidermal SC type in which stabilised β-catenin is expressed, different tumours are formed. For example, targeting the Lgr5^+^ population promotes formation of pilomatricomas (benign HF skin tumours), while Lrig1^+^ cells develop trichoadenomas (a rare benign follicular tumour with cornifying cysts) and the Lgr6^+^ population gives rise to dermatofibromas within the IFE [5]. Similarly, activation of Hedgehog signalling demonstrates that only basal Krt14^+^ cells in the IFE and HF **infundibulum** can initiate basal cell carcinoma (BCC) formation 15, 62. BCC initiation and progression are highly dependent on expression of the transcription factor Sox9 [63]. In contrast, squamous cell carcinoma (SCC) can originate from more than one epidermal population, including HF bulge SCs, as overexpression of a hyperactive Kras mutant in different epidermal lineages induces tumours with comparable efficiency [64]. Consistent with this, it has been shown that SCC cells harbour a distinct open chromatin landscape combining active gene signatures from distinct SC lineages [54]. This state of lineage infidelity can be transiently observed in wounds, but persists during malignant progression, promoting uncontrolled growth and heterogeneous tumour cell behaviour 54, 65.

During tumour initiation, mutant epidermal cells have the ability to engage nontransformed (healthy) cells via paracrine signalling, such as Wnt ligand secretion, to induce aberrant growth of the whole tissue [66]. Intriguingly, the converse is also observed: a tumour protective, ‘neighbourhood watch like’ mechanism, contributes to neoplastic tumour suppression. Using an *in vivo* live imaging approach, it was observed that healthy epithelial cells routinely recognise, surround, and eliminate mutant cells to restore tissue homeostasis, revealing an innate cellular ability to prevent over proliferation and tumour initiation [67]. So far the molecular mechanism of cell recognition and elimination and whether it is restricted to specific cell types remains unclear. The tumour-promoting as well as protective effects upon oncogenic β-catenin signalling rely on Wnt ligand secretion, suggesting that distinct Wnt ligand combinations balance cellular plasticity and behaviour.

## Mesenchymal Heterogeneity in the Tumour Microenvironment

The tumour microenvironment, including the **tumour stroma**, comprising all nontransformed tissue components associated with a tumour, can have both tumour-promoting and -inhibitory effects. Besides endothelial and immune cells a major component of the microenvironment are cancer-associated fibroblasts (CAFs), which play an important role in the evolution of solid tumours. Similar to myofibroblasts, CAFs seem to originate from different mesenchymal populations, ranging from normal fibroblasts and MSCs to transdifferentiated epithelial and endothelial cells. In contrast to normal fibroblasts, CAFs either reside within the tumour margin or infiltrate the tumour mass and show increased proliferation, migration, ECM deposition, and secretion of growth factors and other ECM modulators (reviewed in 68, 69). Functionally, while CAFs are highly heterogeneous in terms of gene expression, they show enrichment of similar **gene ontology** classes such as cell adhesion, immune response and ECM modulation, suggesting that different cell types under similar conditions perform similar tasks. Thus it has been proposed that CAFs represent a dynamic cellular state of fibroblast-like cells in the vicinity of the tumour rather than a specific cell lineage [69]. This state could be maintained by a combination of genetic mutations, epigenetic alterations, and persistent environmental effects.

Similar to wound healing it seems likely that distinct fibroblast populations give rise to CAFs. Indeed, one study identified that in adult mice CD26^+^ fibroblasts are the main contributor to ECM deposition in a skin melanoma xenograft model [25]. Depletion of this fibroblast subpopulation significantly reduced tumour growth, revealing that targeting distinct fibroblast subpopulations impacts tumour development. Inhibition of CD26 activity reduces the growth of wound-induced epidermal tumours, although it must be noted that CD26 is expressed by both epidermal and dermal cells and that during tumour progression there are dynamic changes in dermal CD26 expression [70].

To date there have been few studies of how different fibroblast lineages contribute to tumour stroma formation. Intriguingly, the different tumours induced by stabilizing β-catenin in Lgr5^+^, Lgr6^+^, and Lrig1^+^ epithelial cells exhibit both similarities and differences in stromal composition. In all three cases there are local increases in fibroblast proliferation, ECM remodelling, and expression of CD44, a major hyaluronic acid surface receptor. However, dermal CD26 expression is upregulated in the stroma of Lgr6 but not Lgr5 or Lrig1 tumours. Conversely Lgr6 and Lrig1 tumours have associated stromal inflammation while Lgr5 tumours do not [5]. It will be of interest to discover whether or not these differences reflect differences in the lineages of fibroblasts associated with each type of tumour or whether fibroblasts respond to tumour-specific signals independent of lineage.

## Concluding Remarks

In recent years our appreciation of epidermal and dermal cell heterogeneity has grown, together with a realisation that cells are capable of exhibiting plasticity and changing fate through dedifferentiation and **transdifferentiation**. It is now clear that the responses of the skin to the challenges of wounding or tumorigenesis reflect a combination of changes in cell intrinsic properties and responses to different microenvironments. Microniches and cell memory are exciting new concepts that warrant further investigation, together with unravelling the distinction between cell types and states, for example in tumour stroma (see Outstanding Questions).

As more single cell gene expression profiles become publically available and tools to make those datasets readily accessible to researchers with a biological background improve 19, 71, we anticipate greater appreciation of the significance of cellular heterogeneity. The combination of experimental data with computational modelling 16, 20, 23, 72, 73 not only allows rigorous evaluation of data quality but also fosters hypothesis generation. Ultimately we anticipate major benefits in terms of understanding tissue scale behaviour, dynamics, and the interplay of distinct cell populations during tissue development, homeostasis and disease .

Outstanding Questions How is the behaviour of cell populations in the epidermis and dermis coordinated during wound repair and tumour development on the tissue scale?

What defines and distinguishes cellular state and cellular type in the skin?

What are cell type specific and universal mechanisms for establishing and maintaining cellular identity and plasticity in the skin?

What are the key changes that create a pro-oncogenic environment?

How do distinct epidermal and dermal cell populations contribute to tumour heterogeneity and progression?

What are the dynamics and organisation of microniches in the dermis and epidermis?

## Glossary

**Arrector pili muscle**: smooth muscle attached to the bulge region of the hair follicle that is responsible for piloerection.
**Bulge**: hair follicle region that marks the bottom of the permanent portion of the hair follicle and the insertion point of the arrector pili muscle. The bulge harbours the stem cells of the hair follicle that divide infrequently.
**Cell lineage**: developmental history of a differentiated cell based on the cell from which it arose – an analogy would be a family tree. As cells progress along a lineage they undergo transcriptional and epigenetic changes associated with differentiation. Cells that have a common differentiated state can still arise from different lineages.
**Cellular state**: describes the physiological condition of a given cell defined by molecular profiles, such as transcriptome or proteome. Cell state is distinct from cell lineage and may be transient, such as proliferative or quiescent, or the point of transition from undifferentiated to differentiated.
**Dedifferentiation**: describes the process whereby differentiated cells lose their specialised characteristics and may acquire stem cell potential.
**Dermal papilla**: specialised subset of mesenchymal cells located at the base of each hair follicle. Dermal papilla cells communicate with the neighbouring epithelial cells and provide essential signals for hair follicle formation and regeneration.
**Dermal sheath**: connective tissue sheath consisting of mesenchymal cells that wrap around the hair follicle and span from the bulge to the dermal papilla.
**Gene ontology**: bioinformatic initiative for a unifying representation of gene and gene product attributes across all species. It classifies gene functions according to cellular components, molecular function and biological processes.
**Hair follicle placode**: thickening of the embryonic epidermis that marks the initiation of hair follicle formation.
**Infundibulum**: upper part of the hair follicle, above the entry point of the sebaceous duct, which extends to the interfollicular epidermis.
**Interfollicular fibroblasts**: fibroblasts located between hair follicles.
**Interfollicular epidermis**: multilayered epithelium that forms the outer covering of the skin and is distinct from adnexal structures (hair follicles, sweat glands, and sebaceous glands).
**Isthmus**: hair follicle region between the infundibulum and bulge.
**Junctional zone**: region that lies at the junction between the infundibulum, permanent portion of the hair follicle (bulge) and the sebaceous gland. Also known as the ‘upper isthmus’.
**Lineage tracing**: genetic labelling of single cells or populations with a marker (e.g., fluorescent protein) that is transmitted to all progeny, thus tracking them in space and time.
**Neutral drift model**: holds that cell fate decisions (such as stem cell renewal or differentiation) are governed by stochastic processes.
**Niche**: local environment (microenvironment) of a cell or group of cells that comprises specific extracellular matrix proteins, growth factors, cell–cell interactions, and other signals that regulate cell behaviour.
**Orthokeratotic differentiation**: differentiation programme within the interfollicular epidermis that is characterised by the formation of a granular layer and loss of nuclei in the cornified layers. Characteristic of mouse tail interscale interfollicular epidermis.
**Panniculus carnosus muscle**: layer of striated muscle in the subcutaneous tissue which provides rodent loose skin with twitching and thermoregulation properties and is important for wound contraction.
**Papillary fibroblasts**: subpopulation of dermal mesenchymal cells that are located close to the epidermal basement membrane and surrounded by thin collagen fibres. These fibroblasts are required for hair follicle formation in wounded or reconstituted skin.
**Parakeratotic differentiation**: differentiation programme observed in the scale interfollicular epidermis of mouse tail skin where the nucleus is retained in the cornified layers and the granular layer is absent. Parakeratotic differentiation is a feature of some human skin disorders such as psoriasis.
**Pericyte**: contractile mesenchymal cell that wraps around the endothelial cells associated with small blood vessels in the body.
**Reticular fibroblasts**: subpopulation of dermal mesenchymal cells that are located in the central dermis and surrounded by thick collagen fibres. These cells form the first wave of dermal repair during wound healing.
**Sebaceous gland**: exocrine gland associated with the hair follicle, which secretes sebum to lubricate the skin surface and hair.
**Transdifferentiation**: describes the process of a cell changing its lineage identity.
**Tumour stroma**: microenvironment associated with a tumour comprising all nontransformed tissue components such as extracellular matrix proteins, fibroblasts, immune cells and endothelial cells.
